# Identification of clinical phenotypes and prediction model for the mixed-infection phenotype of pediatric community-acquired pneumonia based on unsupervised machine learning

**DOI:** 10.3389/fped.2026.1785262

**Published:** 2026-05-21

**Authors:** Meng Xiao, Ying Jiang, Qiaobin Chen, Yongxi Deng, Hongbiao Huang, Qiong Fang, Xiaoting Lin, Lijun Xiong

**Affiliations:** 1 Department of Pediatrics, Provincial Clinical Medical College of Fujian Medical University, Fujian Provincial Hospital, Fuzhou University Affiliated Provincial Hospital, Fuzhou, Fujian, China; 2 Department of Pediatrics, Sanming Second Hospital, Sanming, Fujian, China

**Keywords:** community-acquired pneumonia, machine learning, nomogram, pediatric, phenotype classification, prediction model

## Abstract

**Objective:**

Pediatric community-acquired pneumonia (CAP) exhibits significant clinical heterogeneity. Traditional microbiological classification overlooks host factors, making it challenging to accurately determine prognosis and provide targeted, precise treatment. Based on unsupervised machine learning, this study integrates microbiological, host inflammatory response, and clinical characteristics to phenotype pediatric CAP and develops an early prediction model for the Mixed-Infection phenotype.

**Methods:**

A retrospective cohort of 305 pediatric patients with CAP who underwent bronchoalveolar lavage (BAL) was included between November 2022 and October 2025. Using microbiological evidence from BAL fluid, inflammatory markers, and clinical features, k-prototypes clustering was applied to identify and classify phenotypes. A decision tree and nomogram were developed to predict the Mixed-Infection phenotype.

**Results:**

Three clinical phenotypes were identified through machine learning: Mycoplasma-Dominant (37.7%), characterized by Mycoplasma infection with moderate inflammatory response; Mixed-Infection (28.2%), characterized by multi-pathogen coinfection, the youngest age group, and the most extended hospital stays; and High-Inflammation (34.1%), characterized by elevated CRP and WBC levels. The Mixed-Infection phenotype had the highest proportion of prolonged hospitalization (31.4%). However, this difference did not reach statistical significance (*p* = 0.117), suggesting a trend toward higher medical resource utilization that warrants further investigation. A model based on white blood cell count, lactate dehydrogenase, and procalcitonin performed well on the test set (AUC = 0.917, accuracy = 91.3%). The nomogram provided a visual clinical assessment tool for early identification of the Mixed-Infection phenotype.

**Conclusion:**

This study systematically applied k-prototypes clustering to identify three clinical phenotypes, revealing distinct "pathogen-host" interaction patterns among them. We developed a simple early identification tool for the Mixed-Infection phenotype. However, our findings are derived from a bronchoscopy/BAL-selected cohort with more severe or complex disease, which may limit generalizability to all pediatric CAP patients. While this tool shows significant potential, further validation in larger prospective cohorts is needed to confirm its generalizability and clinical applicability.

## 1 Introduction

Community-acquired pneumonia (CAP) remains one of the most common and severe infectious diseases in children worldwide, contributing substantially to morbidity, healthcare utilization, and mortality in children under five years of age (1). Despite advances in vaccination and antimicrobial therapy, the clinical management of pediatric CAP remains a fundamental challenge due to substantial heterogeneity in disease presentation, progression, and treatment response. Children infected with the same pathogen can exhibit markedly different clinical courses—ranging from mild, self-limiting illness to severe, protracted disease requiring intensive care (2, 3). This variability suggests that the pathophysiology of pediatric CAP is not determined solely by the infectious agent but instead emerges from a complex interplay between pathogen characteristics and host immune responses (4).

Traditional approaches to classifying and managing pediatric pneumonia have been predominantly pathogen-centered, relying on microbiological identification or empirical antibiotic coverage targeting likely organisms. However, this “pathogen-determinism” framework has inherent limitations. First, coinfections with multiple pathogens are common in pediatric CAP, and synergistic interactions between viruses and bacteria can amplify inflammatory responses and exacerbate disease severity (5). Second, even in cases of single-pathogen infection (e.g., Mycoplasma pneumoniae), intra-individual differences in host immune responses can lead to divergent inflammatory trajectories and clinical outcomes (6). These observations underscore that a classification system based solely on pathogen type is insufficient to capture the full complexity of pediatric CAP and guide precision treatment.

In recent years, the concepts of “phenotype” and “endotype” have emerged as powerful frameworks for disentangling heterogeneity in complex diseases. In sepsis and adult community-acquired pneumonia, unsupervised machine learning approaches have identified distinct immune-inflammatory phenotypes that differ not only in clinical presentation but also in underlying biology, treatment response, and prognosis (7–10). These findings have important therapeutic implications, suggesting that patients with different phenotypes may benefit from tailored immunomodulatory or anti-inflammatory strategies. However, whether similar phenotype frameworks exist in pediatric CAP—and whether they can inform clinical decision-making—remains largely unexplored. To date, no study has comprehensively phenotyped pediatric CAP using high-quality microbiological evidence from the lower respiratory tract, combined with host inflammatory markers, to capture the full spectrum of pathogen-host interactions.

A significant barrier to phenotype discovery in pediatric CAP has been the lack of integrated data from the lower respiratory tract. Upper respiratory tract specimens (e.g., nasopharyngeal swabs) are susceptible to contamination and may not accurately reflect the pathogen profile in the lungs. Bronchoalveolar lavage fluid (BALF), by contrast, provides a direct window into the lower respiratory tract microenvironment, capturing both the infectious agent(s) and the host's local inflammatory response (11, 12). Systematic phenotyping based on BALF thus offers an unprecedented opportunity to characterize the actual pathogen-host interaction landscape in pediatric CAP.

To address this critical gap, the present study leverages a multicenter cohort of pediatric CAP patients who underwent bronchoscopy with comprehensive BALF microbiological profiling. Using unsupervised machine learning (k-prototypes clustering), we integrate multidimensional data—including BALF microbiological evidence, host inflammatory markers, clinical characteristics, and imaging findings—to achieve the following objectives:

- First, to systematically identify distinct clinical phenotypes of pediatric CAP based on integrated pathogen-host data;
- Second, to characterize the unique pathogen-host interaction patterns associated with each phenotype and their relationship with clinical outcomes;
- Third, to develop and validate clinically translatable predictive models for early identification of the Mixed-Infection phenotype using routine laboratory indicators.

By establishing a phenotype-oriented classification framework grounded in evidence from the lower respiratory tract, we aim to provide a new foundation for precision prevention, control, and individualized treatment of pediatric CAP.

The remainder of this paper is organized as follows: Section 2 describes the study design, patient cohort, and methods; Section 3 presents the results; Section 4 discusses the findings and their implications; and Section 5 concludes the study.

## 2 Methods

### 2.1 Study design and patient cohort

This is a retrospective, multicenter, observational study. We included pediatric patients who were clinically diagnosed with CAP and hospitalized in the Department of Pediatrics at two hospitals in Fujian Province (Fujian Provincial Hospital and Sanming Second Hospital) from November 1, 2022, to October 12, 2025. These patients underwent bronchoscopy and bronchoalveolar lavage.

#### 2.1.1 Inclusion criteria

(1) Clinical assessment upon admission met the diagnostic criteria for pediatric CAP, based on the guidelines from the American Academy of Pediatrics/Infectious Diseases Society of America “Clinical Practice Guidelines for the Management of Community-Acquired Pneumonia in Children" (13), and the 2024 edition of the Chinese Medical Association Pediatric Respiratory Group's diagnostic standards (14).
(2) Age between 3 months and 14 years.
(3) Indications for bronchoscopy and BAL, with family consent obtained.

#### 2.1.2 Exclusion criteria

(1) Age over 14 years.
(2) Severe underlying diseases (e.g., congenital heart disease, primary immunodeficiency, bronchopulmonary dysplasia, etc.).
(3) Incomplete clinical data.

### 2.2 Data collection and feature engineering

The electronic medical record system collected clinical data, laboratory test results, imaging findings, and microbiological detection data. The collected variables included demographic characteristics (age, sex), laboratory inflammatory markers (white blood cell count, neutrophil-to-lymphocyte ratio, lymphocyte-to-neutrophil ratio, C-reactive protein, procalcitonin, lactate dehydrogenase, and D-dimer), imaging features (number of affected lung lobes and significant imaging findings), serum pathogen antibodies, throat swab pathogen PCR, sputum culture, and Next-generation sequencing (NGS) results of bronchoalveolar lavage fluid (BALF). All laboratory parameters used for clustering analysis and model development were the first available measurements obtained at hospital admission (within 24 h of presentation to the emergency department or pediatric ward), and were collected prior to the initiation of antibiotic and/or steroid therapy and before bronchoscopy and bronchoalveolar lavage. This standardized timing ensures that the measured inflammatory markers reflect the baseline host response to infection rather than post-interventional alterations. It aligns perfectly with the clinical scenario of early risk stratification, where such data would be available to guide initial management decisions. In pathogen identification, this study used BALF NGS results as the core basis for diagnosing lower respiratory tract infections, with throat swab PCR, serum pathogen antibodies, and sputum culture results as supplementary criteria. For phenotype analysis, the BALF NGS results were given the highest weight. A pathogen was considered positive if it met any of the following criteria: the primary criterion was a precise detection of the pathogen in BALF NGS (excluding common colonizing bacteria and environmental contaminants); the supplementary criteria were a positive result in throat swab PCR, serum antibody testing, or sputum culture, consistent with clinical manifestations.

The clinical outcome measure was the total length of hospital stay, with a prolonged hospitalization defined as exceeding the 75th percentile of the entire cohort. To construct a feature matrix suitable for clustering analysis, continuous variables were Z-score standardized, and all categorical features were converted to binary variables.

### 2.3 Unsupervised clustering and statistical analysis

Since the study data included continuous and categorical variables, the k-prototypes clustering algorithm was used for unsupervised phenotype identification. In k-prototypes clustering, distance is calculated as the sum of Euclidean distances for continuous variables and simple matching distances for categorical variables, with a balancing parameter *γ* (gamma) that controls the relative contribution of the two variable types. We used the default gamma value automatically estimated by the algorithm, which scales the categorical variable's contribution to be comparable to that of the continuous variable based on the data's inherent variability. No manual feature weighting was applied to prioritize any specific variable or variable type. To determine the optimal number of clusters (k), the range for k was set from 2 to 5, and the clustering algorithm was run for each value. The clustering quality was assessed using the elbow method (evaluating the inflection point of the decrease in within-cluster sum of squares as k increases) and the silhouette coefficient (assessing the compactness of individual samples within their assigned cluster).

After clustering, statistical methods were used to compare baseline characteristics and clinical outcomes across the clusters (i.e., the “phenotypes” defined in subsequent analyses). For continuous variables that followed a normal distribution, one-way analysis of variance (ANOVA) was used; for non-normally distributed variables, the Kruskal–Wallis test was used. Categorical variables were compared using the *χ*^2^ test or Fisher's exact test. All statistical analyses were performed in R (version 4.5.1) with a significance level of *p* < 0.05.

### 2.4 Construction and validation of the prediction model

This study employed a decision tree algorithm to develop a clinical prediction tool for the Mixed-Infection phenotype. The phenotypes identified through clustering analysis were used as the dependent variable, while conventional laboratory markers were the independent variables to construct a classification model. Crucially, all independent variables used in model development were obtained from the same standardized admission blood draw (within 24 h of presentation, prior to treatment), ensuring that the model relies on early, readily available data suitable for accurate early risk stratification, rather than data reflecting post-treatment disease evolution. The dataset was randomly split into a training set and a test set at a 7:3 ratio. The model was trained on the training set using 10-fold cross-validation, with complexity parameters optimized to prevent overfitting. Model performance was evaluated on the independent test set. Discriminatory ability was assessed by plotting the receiver operating characteristic (ROC) curve and calculating the area under the curve (AUC). Additionally, accuracy, sensitivity, and specificity were computed. Finally, based on the final decision tree model, a nomogram was constructed to provide an intuitive visualization of individual patient risk and probability estimation.

### 2.5 Visualization

To comprehensively and intuitively present the clustering results and phenotype characteristics, multiple data visualization techniques were employed: principal component analysis (PCA) scatter plots to illustrate the distribution of phenotypes in the reduced-dimensional space; heatmaps to depict the expression patterns of phenotypes across multidimensional features; radar charts to portray the multidimensional feature profiles of the phenotypes; and box plots and bar charts to compare inter-group differences in clinical outcomes.

## 3 Results

### 3.1 Patient cohort characteristics and phenotype identification

**Figure 1:**
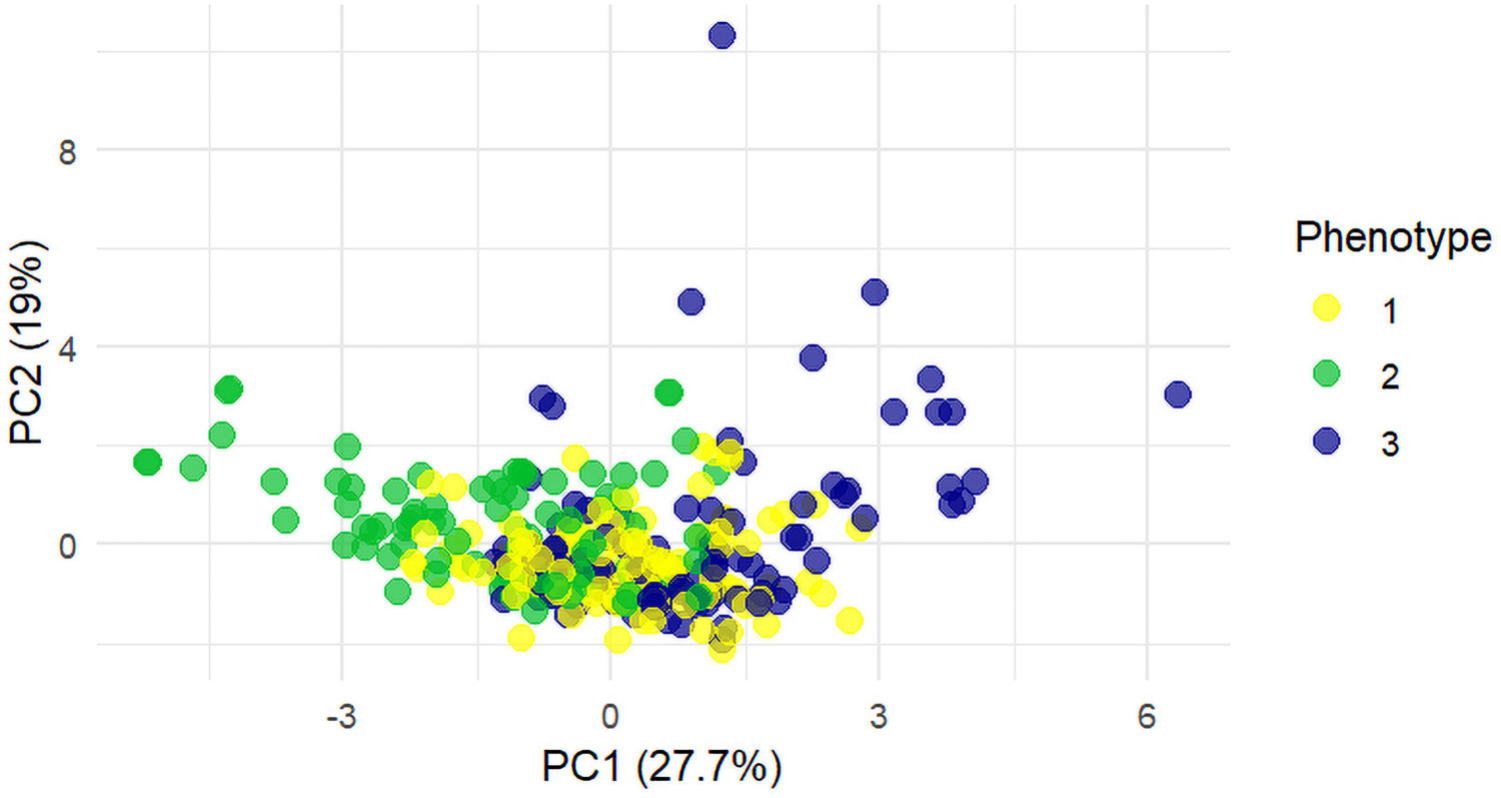
PCA scatter plot of clinical phenotypes in pediatric CAP. PC1 and PC2 explain 27.7% and 19.0% of the total variance, respectively. Different colors or symbols represent different phenotypes: 1 for MP-Dominant, 2 for Mixed-Infection, and 3 for High-Inflammation.

A total of 305 pediatric patients with CAP who underwent bronchoscopy and bronchoalveolar lavage (BAL) were included in this study. Among them, 160 were male (52.5%), and 145 were female (47.5%), with a mean age of 6.7 ± 2.8 years. Unsupervised k-prototypes clustering analysis was performed on 22 clinical features, and the optimal number of clusters (k = 3) was determined using the elbow method and silhouette coefficient. The cohort was divided into three clinically distinct phenotypes: Mycoplasma-Dominant (MP-Dominant) (115 cases, 37.7%), Mixed-Infection (86 cases, 28.2%), and High-Inflammation (104 cases, 34.1%). Principal component analysis (PCA) showed that PC1 and PC2 explained 27.7% and 19.0% of the total variance, respectively. The three phenotypes exhibited a clear separation in the reduced-dimensional space (Figure 1). Notably, the Mixed-Infection phenotype was most clearly separated along the PC1 axis from the other two phenotypes, indicating a unique clinical profile and further supporting the biological plausibility and stability of the clustering results.

### 3.2 Phenotype characteristic spectrum analysis

Significant differences were observed across the three phenotypes regarding demographic characteristics, pathogen composition, inflammatory markers, and imaging findings (Table 1). The MP-Dominant phenotype was predominantly characterized by Mycoplasma infection (72.2%), with relatively older patients (median age 7.0 years) and moderate levels of inflammatory markers (median CRP 10.4 mg/L), suggesting that it may represent the typical clinical group of Mycoplasma pneumonia. The Mixed-Infection phenotype exhibited a complex pattern of multi-pathogen infection, with the highest rates of viral (16.3%) and bacterial (27.9%) infections, and a mixed infection rate of 40.7%. Patients in this group were the youngest (median age 5.0 years), indicating that this phenotype may reflect a high-risk group of younger children exposed to multiple pathogens. The High-Inflammation phenotype was also predominantly driven by Mycoplasma infection (74.0%). However, its distinguishing feature was significantly elevated CRP levels compared to the other phenotypes (median 17.0 mg/L, *p* < 0.001) and also significantly higher WBC counts (*p* = 0.014), indicating a CRP/WBC-driven inflammatory state as measured by routine clinical markers. While this inflammatory profile may have clinical implications, the underlying mechanisms cannot be determined from the available data.

**Table 1:** Comparison of baseline characteristics Among different phenotypes.

Variables	MP-dominant (*N* = 115)	Mixed-infection (*N* = 86)	High-inflammation (*N* = 104)	*p*
Age, Median (Q1, Q3)	7.0 (6.0–9.0)	5.0 (3.0–7.0)	8.0 (6.0–9.0)	<0.001
CRP, Median (Q1, Q3)	10.4 (4.7–17.9)	5.0 (0.9–10.6)	17.0 (4.7–39.3)	<0.001
PCT, Median (Q1, Q3)	0.2 (0.1–0.4)	0.2 (0.1–0.2)	0.2 (0.1–0.3)	0.072
LDH, Median (Q1, Q3)	277 (233–355)	255 (235–300)	260 (211–323.5)	0.200
WBC, Median (Q1, Q3)	6.9 (5.8–8.8)	8.7 (5.9–11.5)	8.2 (6.3–11.5)	0.014
Evidence-MP, *n* (%)	83 (72)	6 (7.0)	77 (74)	<0.001
Evidence-Viral, *n* (%)	3 (2.6)	14 (16)	5 (4.8)	<0.001
Evidence-Bacterial, *n* (%)	5 (4.3)	24 (28)	11 (11)	<0.001
Evidence-Mixed, *n* (%)	24 (21)	35 (41)	11 (11)	<0.001
CT-GGO, *n* (%)	3 (2.6)	9 (10)	2 (1.9)	0.014
CT-Consolidation, *n* (%)	102 (89)	68 (79)	85 (82)	0.200
Prolonged-LOS, *n* (%)	22 (19%)	27 (31%)	23 (22%)	0.120

CT-GGO, CT ground-glass opacity; prolonged-LOS, prolonged length of stay (defined as hospitalization > 10 days).

**Figure 2:**
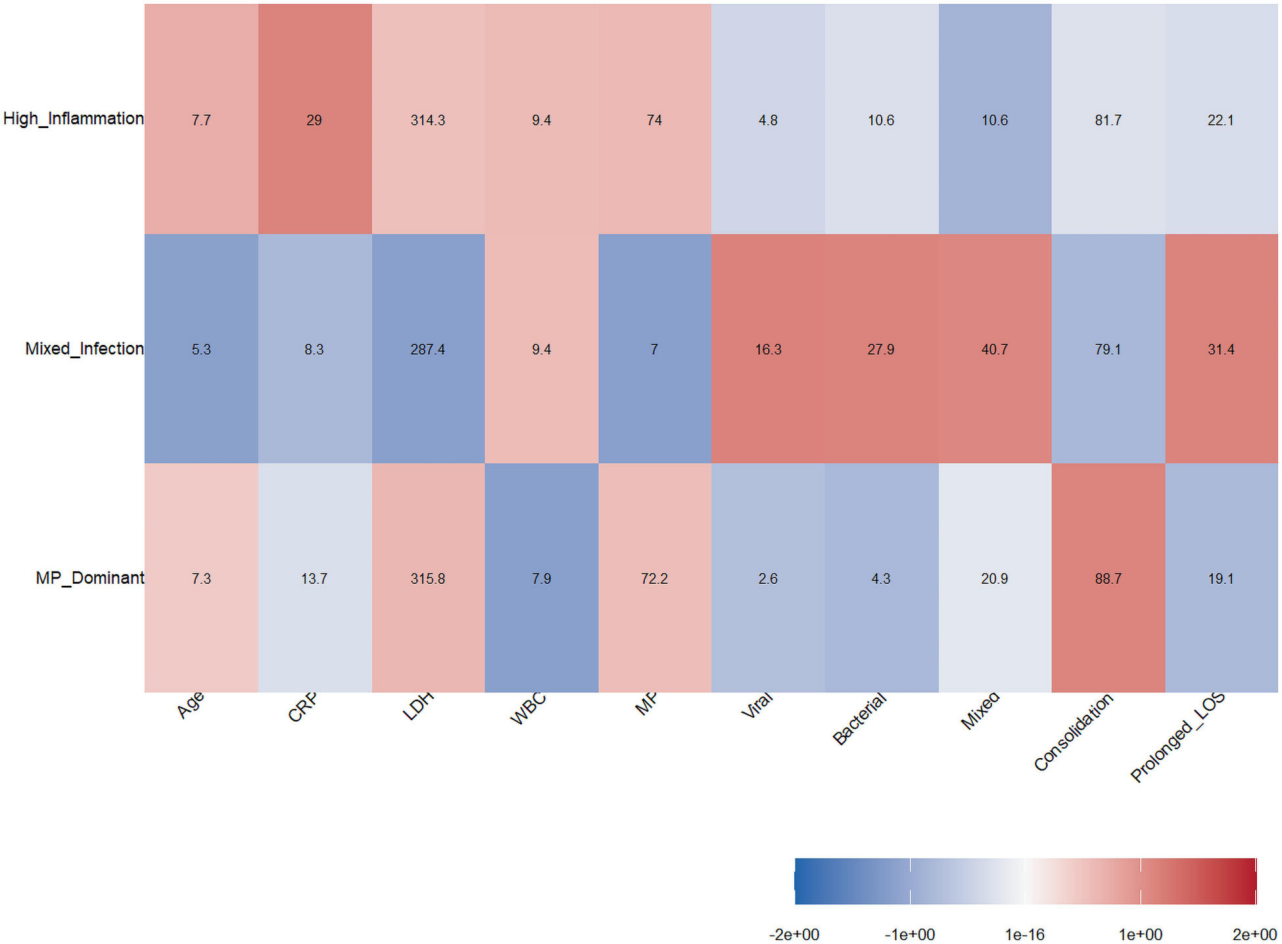
Feature heatmap of three clinical phenotypes in pediatric CAP. The horizontal axis shows clinical features, and the vertical axis shows the three clinical phenotypes.

**Figure 3:**
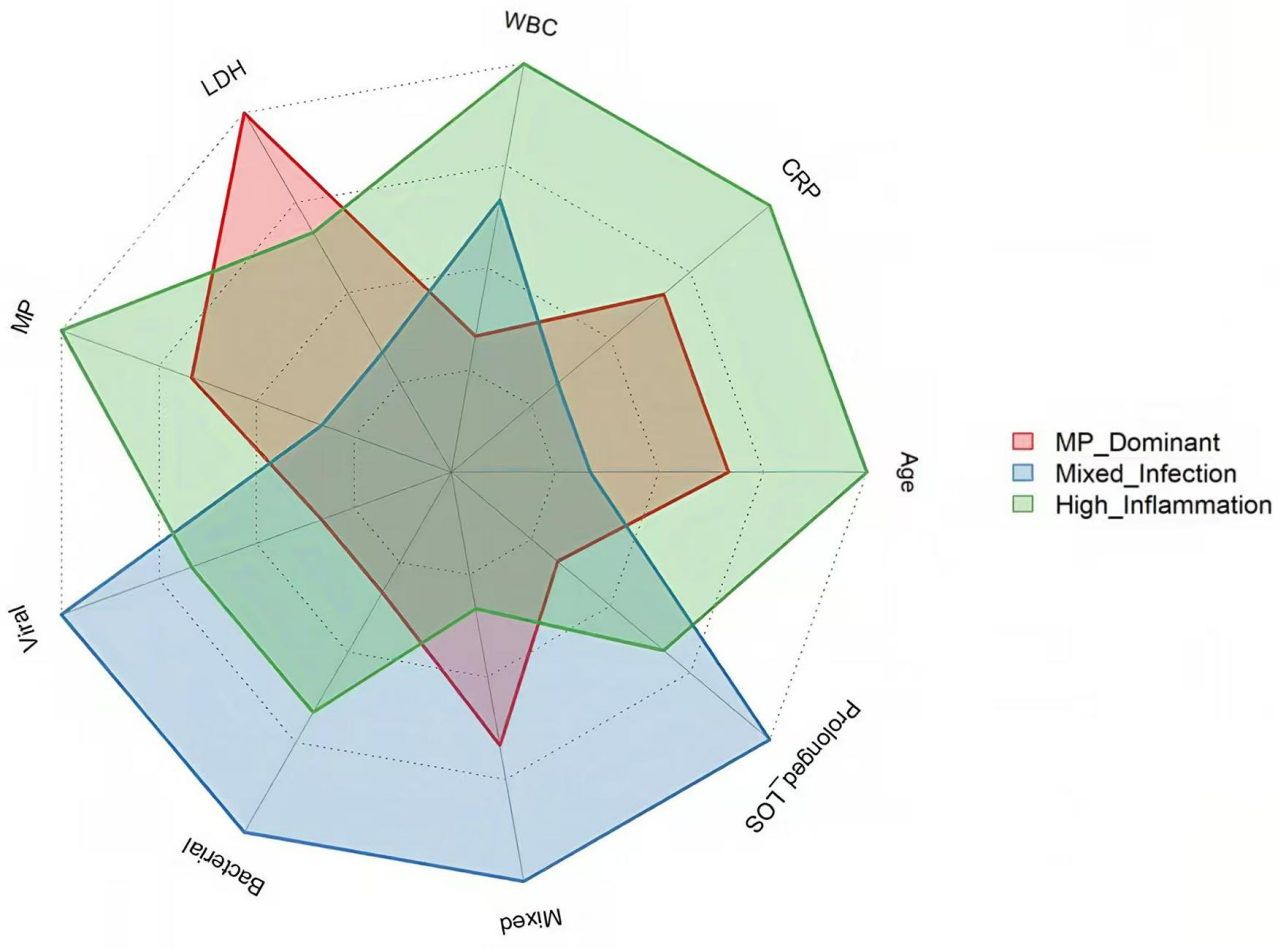
Radar chart analysis of the feature profiles of three clinical phenotypes. Each axis represents a clinical feature. The polygon's area and shape reflect the composite characteristic profiles of each phenotype.

The heatmap analysis visually presents the differential patterns of the three clinical phenotypes in the multidimensional feature space (Figure 2). The Mixed-Infection phenotype showed the highest Z-scores for characteristics such as mixed infection (40.7%), bacterial infection (27.9%), and viral infection (16.3%), reflecting its typical feature of multi-pathogen coinfection. In contrast, the MP-Dominant and High-Inflammation phenotypes both exhibited high values for Mycoplasma infection indicators (72.2% and 74.0%, respectively), suggesting that both are primarily driven by Mycoplasma infection, but with significant differences in the intensity of the inflammatory response. The heatmap revealed that the Mixed-Infection phenotype had a higher proportion of prolonged hospitalizations (31.4%), suggesting it may be associated with a more complex clinical course. Radar chart analysis (Figure 3) further depicted the composite characteristic profiles of the three clinical phenotypes regarding morphological and structural dimensions. The MP-Dominant phenotype exhibited a concentrated pattern centered on Mycoplasma infection, whereas the Mixed-Infection phenotype displayed a broad spectrum of pathogen distribution, including viruses, bacteria, and mixed infections. On the other hand, the High-inflammatory phenotype was prominently highlighted along inflammatory response axes, including white blood cells and CRP, suggesting a strong systemic inflammatory state.

**Figure 4:**
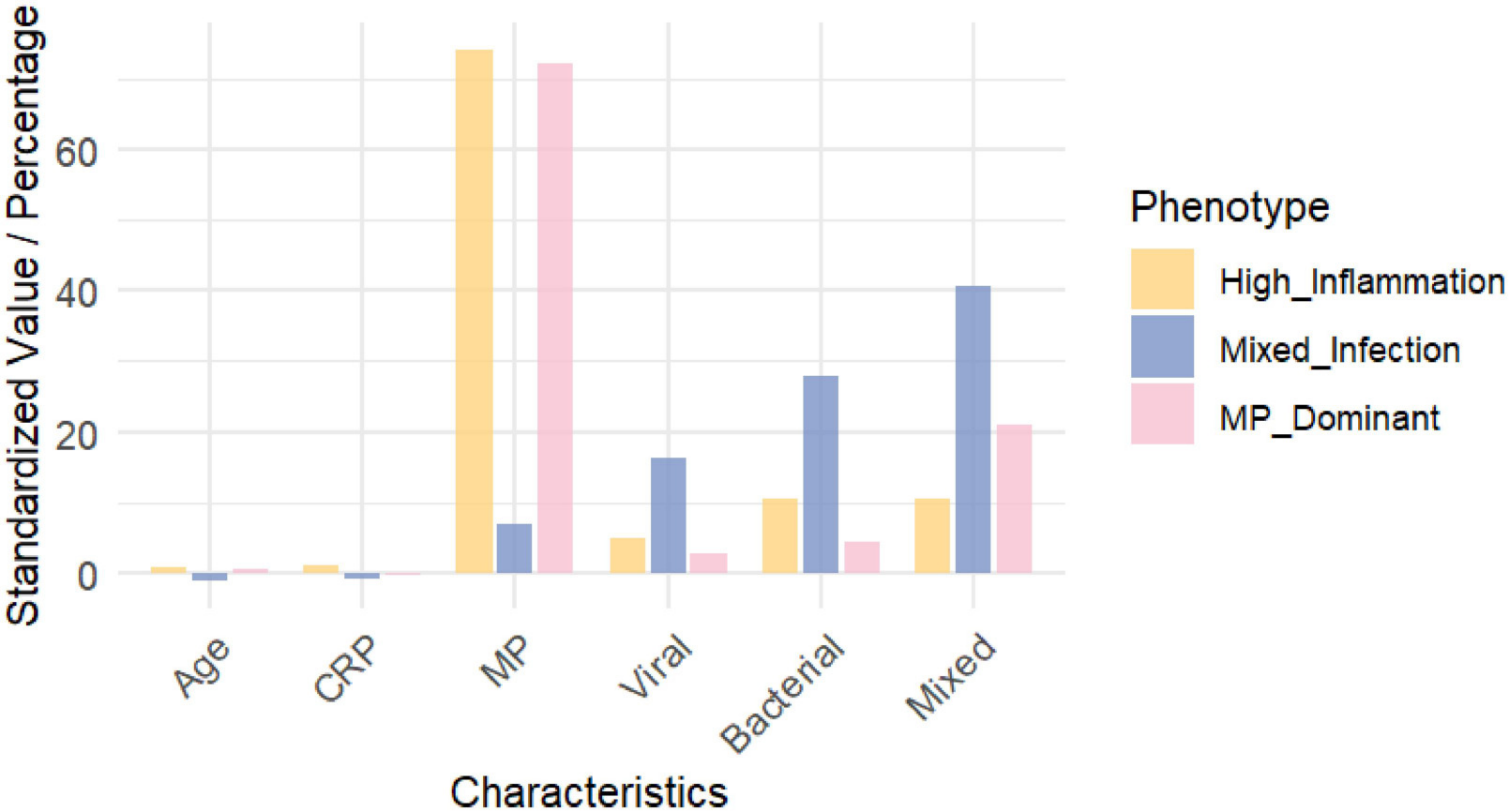
Bar chart of Key feature distributions for three clinical phenotypes. The horizontal axis represents the feature variables, and the vertical axis represents the standardized values or percentages.

The bar chart of key feature comparisons (Figure 4) further quantitatively validates the significant differences in core indicators among the clinical phenotypes, highlighting the roles of inflammatory response levels and pathogen composition in phenotype differentiation. The chart clearly shows that the Mixed-Infection phenotype exhibits significantly higher values than the other phenotypes in mixed infection (40.7%), bacterial infection (27.9%), and viral infection (16.3%), forming its unique “multi-pathogen coinfection” feature profile. Meanwhile, the High-Inflammation phenotype's prominent performance in CRP levels (median 17.0 mg/L) aligns well with its name, reflecting a strong systemic inflammatory response characteristic. In contrast, the MP-Dominant phenotype clearly dominates Mycoplasma infection indicators (72.2%), indicating a typical single-pathogen-dominant pattern. These differential patterns statistically reinforce the validity of the phenotype classification and provide key features for the subsequent development of the prediction model.

### 3.3 Association analysis of phenotypes and clinical outcomes

**Figure 5:**
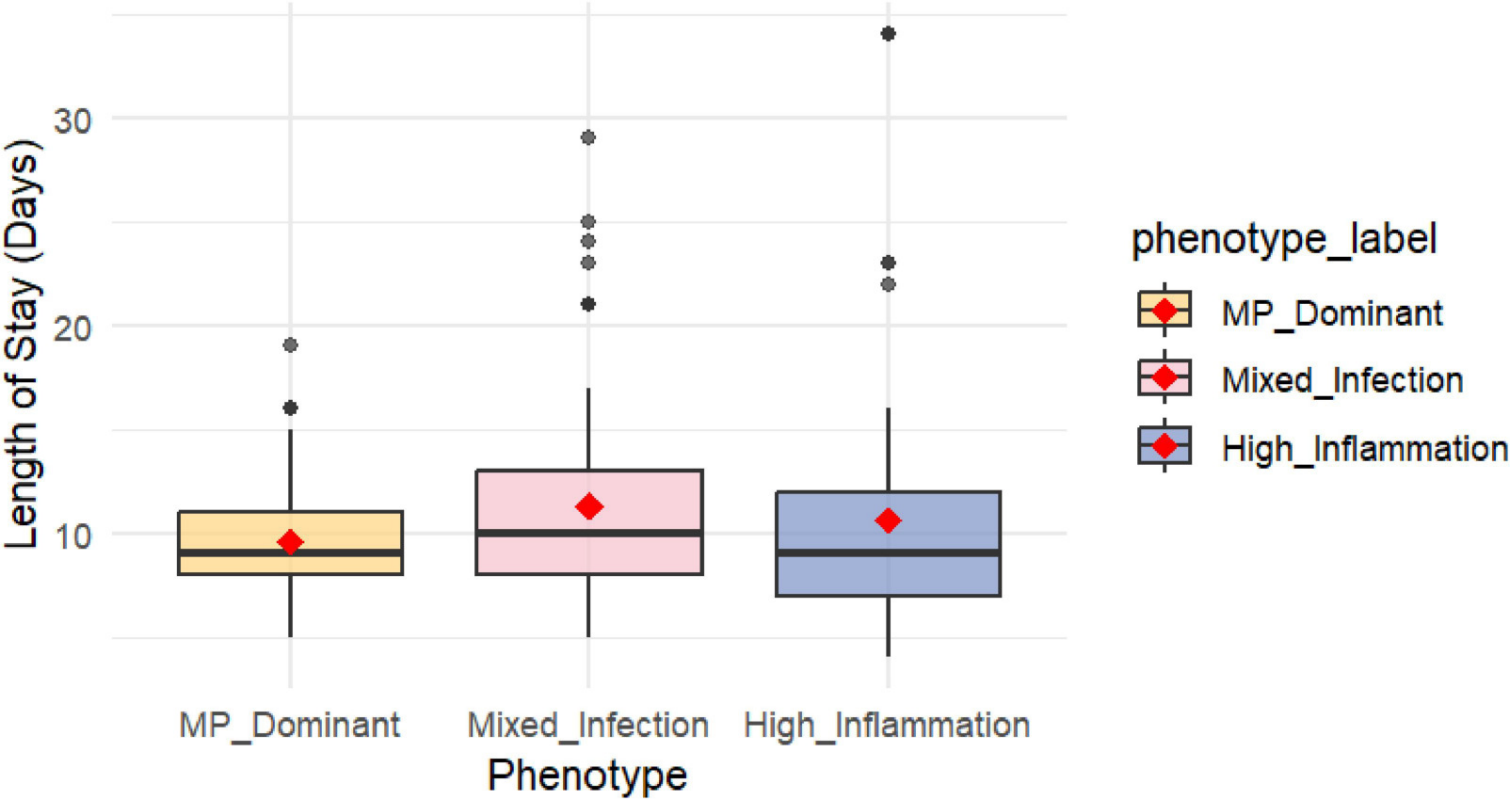
Box plot comparison of hospitalization duration distribution across three clinical phenotypes.

The phenotypes exhibited clinically significant differences in clinical outcome measures (Table 2). Regarding hospitalization duration, the Mixed-Infection phenotype had the most extended median hospital stay (10.0 days), slightly longer than those of the MP-Dominant and High-Inflammation phenotypes (both 9.0 days). This difference did not reach statistical significance (*p* = 0.089). The observed trend, while not definitive, suggests a possible longer recovery course in the Mixed-Infection phenotype that warrants confirmation in larger studies (Figure 5). The box plot in Figure 5 visually shows the distribution of hospitalization duration for each phenotype. The box represents the interquartile range (IQR), and the line inside indicates the median. The Mixed-Infection phenotype has a broader distribution of hospitalization durations, with the highest median hospitalization duration. Its IQR (8.0–13.0 days) is skewed toward higher values compared with those of the other phenotypes, suggesting that patients in this phenotype generally have more extended hospital stays. Moreover, the Mixed-Infection phenotype shows a higher number of outliers exceeding the upper limit, further indicating that some patients in this phenotype experienced prolonged hospital stays, potentially reflecting a more complex clinical course.

**Table 2:** Comparison of clinical outcomes among different clinical phenotypes.

Outcome	MP-dominant (*N* = 115)	Mixed-infection (*N* = 86)	High-inflammation (*N* = 104)	*p*
Length of Stay, Median (Q1, Q3)	9.0 (8.0–11.0)	10.0 (8.0–13.0)	9.0 (7.0–12.0)	0.089
Prolonged Hospital Stay, *n* (%)	22 (19.1)	27 (31.4)	23 (22.1)	0.117
Complications, *n* (%)	21 (18.3)	28 (32.6)	36 (34.6)	0.045
Poor Clinical Outcome, *n* (%)	0 (0.0)	1 (1.2)	0 (0.0)	0.279

Data are presented as median (interquartile range) or frequency (percentage). Prolonged hospitalization is defined as a hospital stay exceeding the 75th percentile (>10 days).

**Figure 6:**
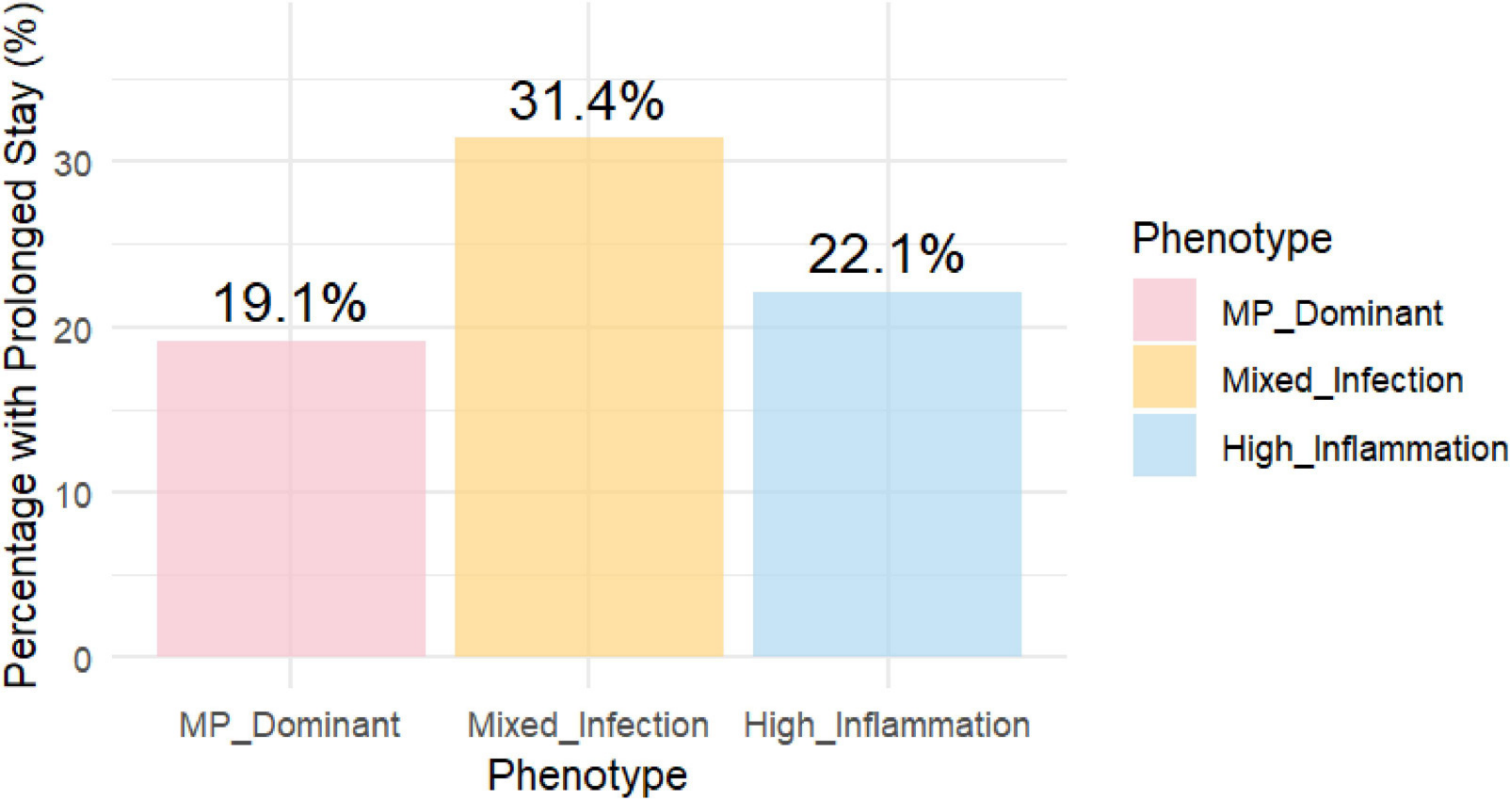
Comparison of the proportion of prolonged hospitalization across three clinical phenotypes.

Further analysis of clinical outcomes revealed distinct patterns across phenotypes. The Mixed-Infection phenotype had the highest proportion of prolonged hospitalization (31.4%), compared to the MP-Dominant (19.1%) and High-Inflammation (22.1%) phenotypes (Figure 6). This difference was not statistically significant (*p* = 0.117); therefore, the association with prolonged hospitalization should be interpreted as a non-significant trend requiring validation in larger cohorts. In contrast, complication rates (including pleural effusion, necrotizing pneumonia, and atelectasis requiring intervention) differed significantly across groups: the High-Inflammation phenotype exhibited the highest complication rate (34.6%), followed by the Mixed-Infection (32.6%) and MP-Dominant (18.3%) phenotypes (*p* = 0.045; Table 2). Notably, the Mixed-Infection phenotype showed a significantly higher complication burden compared to the MP-Dominant phenotype (32.6% vs. 18.3%), providing statistically robust evidence of its clinical importance. These findings suggest that while the association with prolonged hospitalization requires confirmation in larger studies, the Mixed-Infection phenotype is associated with a clinically meaningful increase in complications, supporting its relevance for early identification and targeted management.

### 3.4 Development of a prediction model for the mixed-infection phenotype

**Figure 7:**
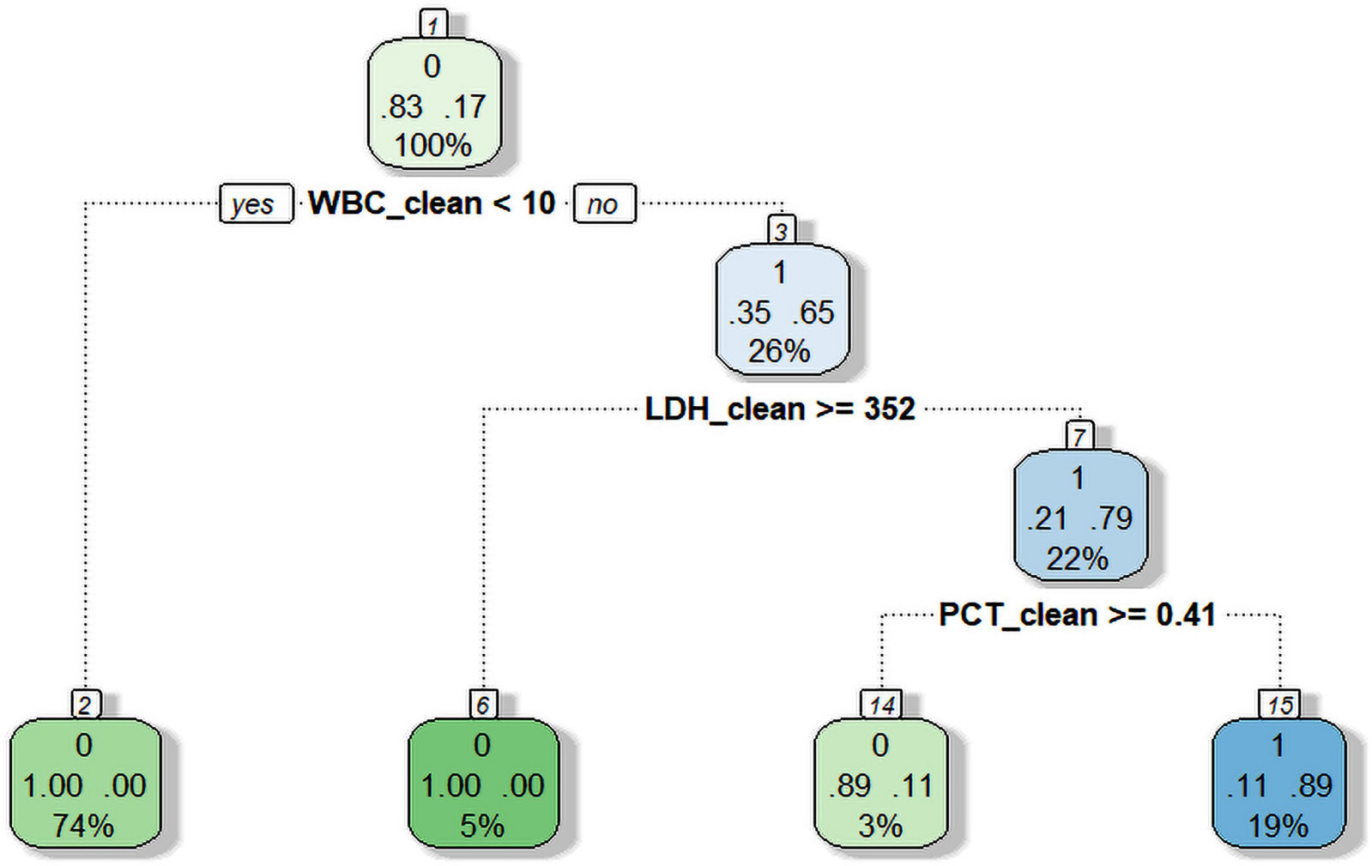
Decision tree model for predicting the mixed-infection phenotype.

Given that the Mixed-Infection phenotype exhibited the most complex clinical characteristics and the highest complication burden, we further developed an early identification tool for this phenotype to facilitate timely recognition and potential targeted management. Based on routine laboratory indicators, a decision tree model constructed using machine learning methods identified three key predictive factors: white blood cell count (WBC), lactate dehydrogenase (LDH), and procalcitonin (PCT). Decision tree analysis (Figure 7) showed that when a child met the criteria of WBC ≥ 10.5 × 10^9^/L, LDH < 351.5 U/L, and PCT < 0.406 ng/mL, the probability of being classified into the Mixed-Infection phenotype was as high as 89.5%. This decision rule has straightforward clinical utility, providing a practical tool for early identification of complex infection phenotypes.

**Figure 8:**
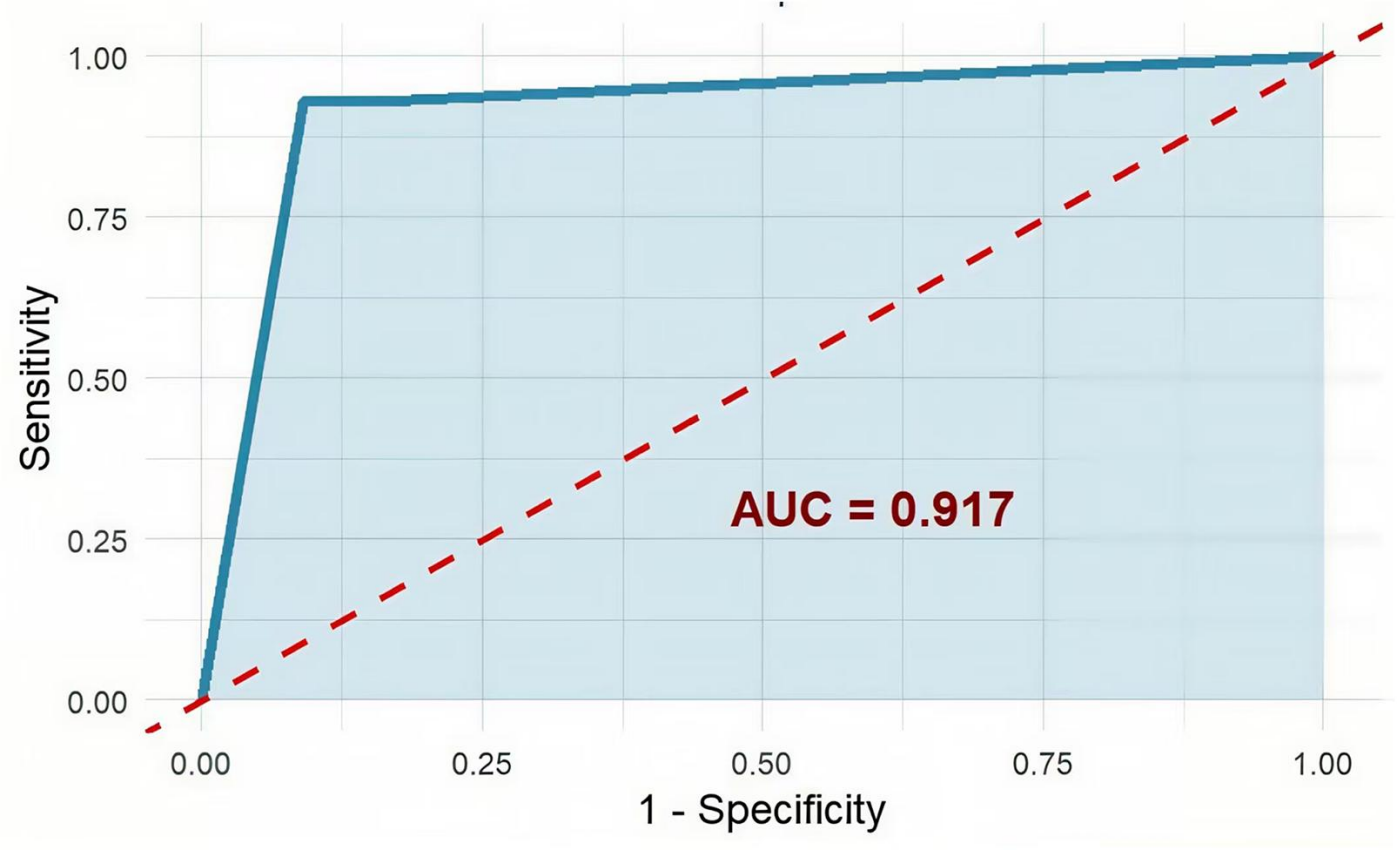
ROC curve for predicting the mixed-infection phenotype. The horizontal axis represents 1-specificity, and the vertical axis represents sensitivity. The diagonal, gray-dashed line indicates random prediction performance.

We performed internal validation using a training-test split method to validate the model's generalizability. The model demonstrated excellent predictive performance in the independent test set (*n* = 92), with an accuracy of 91.3% (95% CI: 83.6%–96.2%), sensitivity of 92.9% (95% CI: 66.1%–99.8%), and specificity of 91.0% (95% CI: 82.4%–96.3%). ROC curve analysis showed the model's strong discriminatory ability, with an AUC of 0.917 (95% CI: 0.852–0.982) (Figure 8). Ten-fold cross-validation further confirmed the model's stability, with an average AUC of 0.952 (standard deviation 0.031).

**Figure 9:**
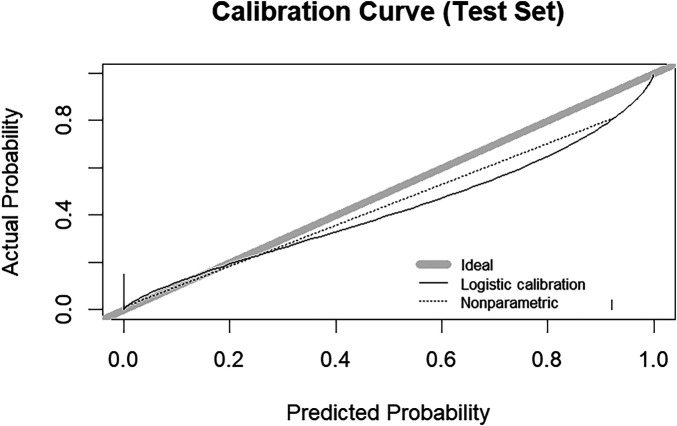
Calibration curve for the prediction model in the test set.

Calibration analysis was performed on the test set to assess the agreement between predicted probabilities and observed outcomes for the Mixed-Infection phenotype. As shown in Figure 9, the calibration curve demonstrated good agreement, with a calibration intercept of −0.41 (close to ideal 0), a calibration slope of 0.74 (close to ideal 1), a mean absolute error of 0.031, and a maximum absolute error of 0.114. These metrics indicate that the model's predicted probabilities are well-calibrated and clinically reliable.

**Figure 10:**
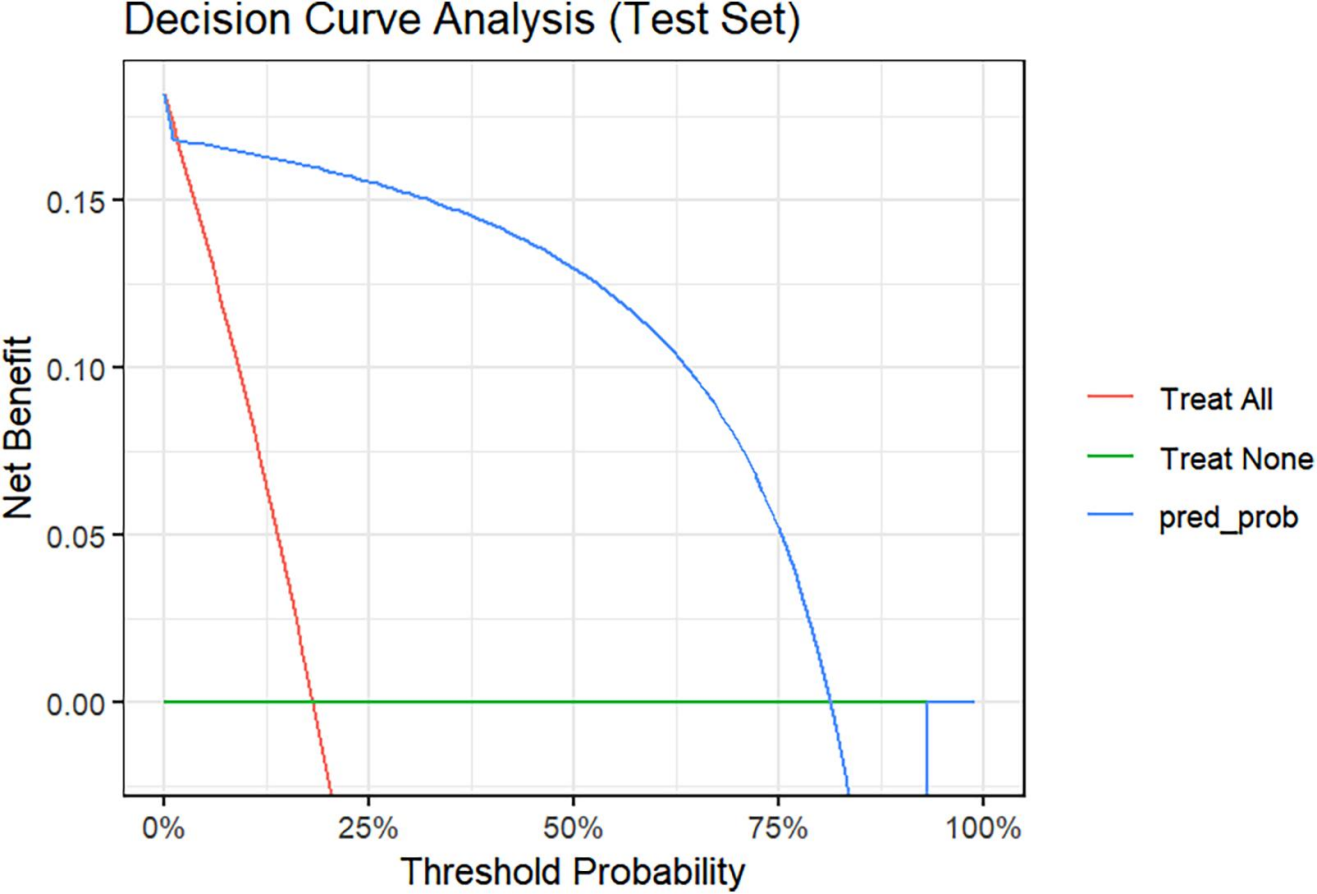
Decision curve analysis for the prediction model in the test set.

Decision curve analysis was performed to evaluate the model's clinical utility. As shown in Figure 10, across a threshold probability range of 2% to 81%, the model demonstrated a higher net benefit than both “treat-all” and “treat-none” strategies. This suggests that using the model to guide early identification of the Mixed-Infection phenotype would provide clinical benefit across most clinically relevant decision thresholds.

**Figure 11:**
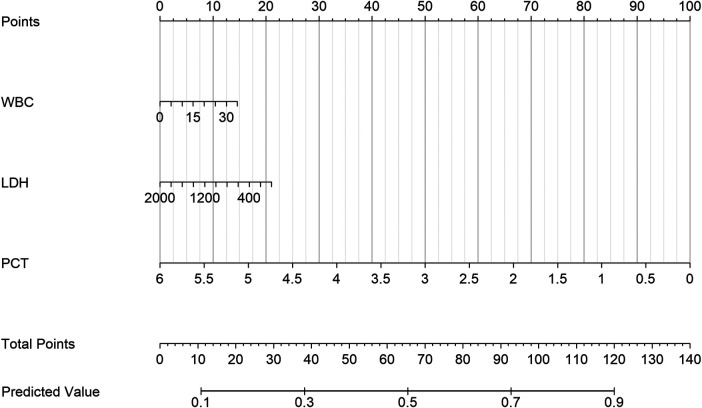
Nomogram for predicting the mixed-infection phenotype. Based on the decision tree model, this nomogram predicts the probability of a child belonging to the Mixed-Infection phenotype. Use the measured values of WBC, LDH, and PCT to determine points on the corresponding axes, sum them to get Total Points, and find the predicted probability on the Predicted Value axis.

Based on the prediction model, we developed a clinically practical nomogram (Figure 11) that enables clinicians to quickly estimate the probability that a child belongs to the Mixed-Infection phenotype using a simple scoring system. This tool requires only three routine test indicators, making it easy to implement in clinical settings.

### 3.5 Significance and translational application of phenotype characteristics

Based on the above analysis, the three pneumonia phenotypes identified in this study exhibit distinct clinical and microbiological characteristics, closely related to the intensity of inflammatory response, treatment response, and medical resource consumption. The MP-Dominant phenotype represents a typical Mycoplasma pneumonia group with relatively simple pathogens and a moderate inflammatory response. This phenotype responds well to targeted therapy, has the best prognosis, and may benefit from standardized Mycoplasma treatment regimens. The Mixed-Infection phenotype reflects the complex clinical state of multi-pathogen coinfection, with a non-significant trend toward longer disease course and potentially higher medical resource consumption (*p* = 0.089 for LOS, *p* = 0.117 for prolonged hospitalization). These trends, together with the significantly higher complication rate (*p* = 0.045), suggest that these patients may benefit from a more comprehensive anti-infection strategy and supportive treatment. However, given the lack of statistical significance for length-of-stay outcomes, these associations should be interpreted cautiously and validated in future studies with larger sample sizes. The predictive model developed in this study provides an effective tool for the early identification of this phenotype. The High-Inflammation phenotype, characterized by elevated routine inflammatory markers at admission, may be associated with a more intense inflammatory response. Based on the available data (limited to routine clinical markers without direct immunological measurements), this phenotype should be interpreted as reflecting a pronounced systemic inflammatory state rather than confirmed immune dysregulation. Clinically, this inflammatory profile may warrant consideration of anti-inflammatory therapy, although the optimal approach requires further investigation.

## 4 Discussion

This study used unsupervised machine learning analysis on 305 pediatric patients with community-acquired pneumonia who underwent bronchoalveolar lavage, revealing three clinically distinct phenotypes. More importantly, this study represents an initial effort to translate a theoretical description of phenotype analysis into a clinical tool. We established a phenotype framework related to prognosis. We successfully developed a predictive model and nomogram for the early identification of the Mixed-Infection phenotype, providing key tools for the precise management of pediatric pneumonia.

From a pathophysiological perspective, the three phenotypes identified in this study map to three distinct “pathogen-host” interaction patterns. The MP-Dominant phenotype exhibits the typical “single pathogen-moderate inflammation” characteristics, with its relatively good prognosis closely linked to the specific pathological mechanisms of Mycoplasma pneumonia. Existing studies have shown that Mycoplasma primarily adheres to respiratory epithelial cells via the P1 adhesion protein on its surface and releases the community-acquired respiratory distress syndrome toxin, inducing epithelial cell damage and ciliary dysfunction (15–17). This relatively localized infection pattern may explain the moderate inflammatory response and relatively mild clinical course in children with this phenotype. Notably, our findings align with the global understanding of the heterogeneity of respiratory infections (18, 19). However, in pediatric pneumonia, we provide systematic evidence for the independent existence of this phenotype based on high-quality bronchoscopy microbiological evidence.

The Mixed-Infection phenotype demonstrates the “immune-inflammation cascade amplification” effect caused by multi-pathogen coinfection. Viral infections compromise the integrity of the respiratory epithelial barrier and downregulate the host's innate immune response, creating a microenvironment for bacterial adhesion, colonization, and invasion. Secondary bacterial infections further activate a strong inflammatory response, forming a positive feedback loop that ultimately leads to more complex clinical manifestations and prolonged recovery (20–23). Children with this phenotype are generally younger, which is closely related to the physiological characteristics of underdeveloped immune systems, incomplete mucosa-associated lymphoid tissue function, and limited specific immune response ability in infants and young children. Although the differences in length of stay did not reach statistical significance (*p* = 0.089 for median LOS, *p* = 0.117 for prolonged hospitalization), the consistently higher complication rate (*p* = 0.045) supports the clinical relevance of this phenotype. This finding provides important theoretical support for early intervention strategies in younger patients.

The High-Inflammation phenotype, characterized by elevated routine inflammatory markers (CRP and WBC) at admission, represents a group of children with a pronounced systemic inflammatory response despite a microbiological profile similar to the MP-Dominant phenotype (predominantly Mycoplasma). While this phenotype suggests heterogeneity in host response intensity, the mechanisms underlying this heightened inflammatory state cannot be determined from our data, as direct immunological measurements (e.g., cytokines, immune cell profiling) were not performed. Notably, the limited differences in PCT and LDH across phenotypes (Table 1) further underscore that the characterization of this phenotype is primarily driven by CRP and WBC, rather than a broad panel of inflammatory markers. This is why we describe the phenotype as “High-Inflammation” based on the two most clinically relevant indicators, while explicitly noting that other inflammatory markers did not reach statistical significance. Previous studies have suggested that severe inflammatory responses in Mycoplasma pneumonia may involve mechanisms such as Toll-like receptor pathway activation, Th1/Th2 imbalance, and elevated pro-inflammatory cytokines (IL-6, IL-8, TNF-α) (24, 25). However, whether these mechanisms underlie the High-Inflammation phenotype identified in our study remains speculative, as our data are limited to routine clinical markers. This excessive inflammatory response, regardless of its underlying mechanisms, may be associated with more severe clinical manifestations and prolonged recovery and has been linked to complications such as coagulation system activation and endothelial dysfunction in previous studies (26, 27). Future studies incorporating comprehensive immune profiling are needed to elucidate the biological basis of this phenotype and determine whether it represents a qualitatively distinct host response or simply a quantitative difference in inflammatory marker elevation. Our findings are consistent with the latest international understanding of the inflammatory response characteristics of severe pneumonia (28, 29). However, the unique aspect of this study lies in the collection of microbiological evidence from bronchoalveolar lavage fluid, which provides a more robust characterization of the infectious context associated with this inflammatory phenotype.

The main contribution of this study is the successful translation from “phenotype identification” to “clinical prediction”. The association analysis between phenotype characteristics and clinical outcomes indicates that the impact of phenotypes on prognosis is mediated by specific features, such as their intrinsic pathogen composition and inflammatory levels, providing the theoretical foundation for developing simplified prediction tools. We constructed a decision tree model and nomogram using three routine indicators (WBC, LDH, and PCT). Importantly, all three indicators were measured at admission prior to any therapeutic interventions, confirming that the model genuinely reflects early risk stratification rather than post-treatment disease evolution. This design choice enhances the model's clinical utility by providing actionable information during the critical window when initial management decisions are being made. The model demonstrated excellent discriminatory performance in the independent test set (AUC: 0.917). We developed a clinical decision-making tool based on the phenotype concept, using routine indicators, that provides a practical, visual aid for clinicians. This tool enables early screening for the high-burden Mixed-Infection phenotype, providing a valuable time window for timely adjustments to treatment plans (e.g., expanding pathogen coverage or enhancing supportive therapy).

The results of this study have clear implications for clinical practice. It advances the management strategy for pediatric pneumonia from the traditional “pathogen-oriented” approach to a more “phenotype-oriented” precision model. For the MP-Dominant phenotype, standard Mycoplasma-targeted treatment may be sufficient; for the Mixed-Infection phenotype, a more comprehensive anti-infection regimen should be initiated; and for the High-Inflammation phenotype, the need for combining immune-regulatory therapy with anti-infection treatment is highlighted. The predictive model and nomogram developed in this study serve as a key bridge for translating this precision medicine concept into clinical application.

This study should be viewed with an awareness of its limitations. First, our cohort included only children with CAP who underwent bronchoscopy and BAL, a population typically characterized by more severe or complex disease, failed initial antibiotic therapy, or suspected complications. This selection bias has three important implications. First, compared to the general CAP population, our cohort likely enriches for Mixed-Infection and High-Inflammation phenotypes, as these are associated with greater clinical complexity. Consequently, the prevalence estimates reported here may not reflect those in milder, community-managed cases, where the MP-Dominant phenotype might be more common. Second, this selection bias may overestimate the clinical burden and resource utilization associated with complex phenotypes, but it does not invalidate the biological distinctions observed among the three phenotypes, as these distinctions are grounded in pathogen coinfection patterns, age distribution, and inflammatory marker profiles—factors that are not artifacts of selection. Third, whether these phenotypes can be reliably identified in non-bronchoscoped patients using less invasive sampling (e.g., nasopharyngeal swabs combined with serum markers) remains an open question that requires dedicated validation in future prospective studies that include a broader spectrum of CAP severity. We have therefore framed our conclusions as applicable primarily to children with moderate-to-severe CAP who undergo bronchoscopy, with cautious extrapolation to the broader pediatric CAP population pending external validation.

Although based on high-quality bronchoscopy microbiological evidence, the retrospective design and limited sample size may affect the analytical power for specific subgroups, and the model's predictive performance still requires external validation in large prospective cohorts. We also acknowledge the potential for optimism bias given the moderate sample size (*n* = 305) and internal validation design. The difference between cross-validation AUC (0.952) and test set AUC (0.917) reflects some degree of optimism, which is expected in datasets of this size. However, the test set AUC of 0.917 remains excellent, and the model's simplicity (three-variable decision tree) reduces the risk of severe overfitting. Moreover, the calibration analysis (calibration intercept: −0.41, slope: 0.74, mean absolute error: 0.031) and decision curve analysis (positive net benefit across the 2%–81% threshold range) provide additional evidence that the model is clinically meaningful beyond mere discrimination.

Additionally, we acknowledge a potential methodological concern regarding partial circularity: some variables used for phenotype clustering (e.g., inflammatory markers such as CRP and WBC) are conceptually related to disease severity. In contrast, prolonged length of stay—a severity-related outcome—was used to validate the clinical relevance of the identified phenotypes. This is an inherent challenge in phenotype discovery studies, as clustering aims to group patients based on their baseline characteristics, and validating these groups against meaningful clinical outcomes is essential to demonstrate their utility. However, to minimize this concern, our clustering approach integrated a broad range of features beyond severity-related markers, including comprehensive microbiological evidence from BALF, demographic characteristics, and imaging findings. The resulting phenotypes therefore reflect multidimensional host-pathogen interaction patterns rather than being driven solely by severity indicators. To explicitly test whether the three phenotypes were driven solely by inflammatory markers, we performed a sensitivity analysis that excluded all inflammatory markers (PCT, CRP, LDH, D-dimer, WBC, neutrophil and lymphocyte ratios) and retained only microbiological, demographic, and imaging features. The optimal number of clusters remained three, and the resulting clusters were distinguished primarily by pathogen composition and age (Supplementary Table S2). The two original MP-rich phenotypes (MP-Dominant and High-Inflammation) largely merged without inflammation markers, confirming that their separation is mainly driven by CRP/WBC. The Mixed-Infection phenotype remained distinct in microbial profile. This analysis demonstrates that the three-phenotype structure is not an artifact of including severity-related markers but reflects genuine pathogen-host interaction patterns.

Furthermore, the current phenotypes are primarily based on clinical and routine laboratory indicators, without integrating molecular features such as pathogen load or host transcriptomics. These deeper layers of information would help further refine the phenotypes and reveal their biological essence. External validation of the model's generalizability in independent, multicenter cohorts is urgently needed before clinical implementation and is a priority for our ongoing research.

To translate phenotype identification into clinical practice, future research should move beyond association to causation. Specifically, we plan to conduct target trial emulation (TTE) using large-scale observational databases, strictly following the step-by-step framework proposed by Yang et al. (30). We will emulate a pragmatic randomized trial that assigns patients with different phenotypes to phenotype-specific management strategies vs. standard care. Outcomes of interest will include time to clinical stability, complication rate, and length of stay. Such TTE analyses would provide causal evidence on whether phenotype-guided therapy improves patient-important outcomes, filling the current translational gap.

We also recognize that pediatric CAP is a dynamic condition with substantial inter- and intra-individual variability in disease trajectories. Our static phenotyping at admission may not fully capture the temporal evolution of host-pathogen interactions. Future work should integrate longitudinal sampling with trajectory modeling methods such as group-based trajectory modeling or latent growth mixture modeling, as recommended. These approaches could identify dynamic endotypes that better inform time-sensitive treatment decisions (31).

Future studies should externally validate the model's generalizability in multicenter prospective cohorts, incorporate additional outcome measures beyond complications (e.g., ICU admission, mechanical ventilation, antibiotic regimen comparisons), and deepen the understanding of phenotype heterogeneity by integrating molecular immunology and pathogen load data. The present study did not assess differences in antibiotic therapy across phenotypes, nor did it capture ICU admission or ventilation requirements; these important clinical endpoints should be prioritized in future prospective investigations to establish the clinical utility of phenotype-guided management fully.

This study systematically describes the three core clinical phenotypes of pediatric community-acquired pneumonia and their biological underpinnings. More importantly, it develops and validates an efficient, practical clinical prediction model. This achievement represents a step toward shifting the focus of pediatric pneumonia research from ‘describing heterogeneity’ to ‘predicting individual risk,’ contributing to the methodological foundation for a phenotype-oriented approach with potential clinical translational value.

## 5 Conclusion

Based on bronchoalveolar lavage microbiological evidence and unsupervised machine learning, this study identified three core phenotypes of pediatric community-acquired pneumonia: MP-Dominant, Mixed-Infection, and High-Inflammation. These phenotypes correspond to three distinct patterns: "single pathogen-moderate inflammation", "multi-pathogen coinfection", and "pronounced systemic inflammatory response based on routine laboratory markers", respectively. The predictive model developed using routine indicators can early identify the Mixed-Infection phenotype, which demonstrated a trend toward more complex clinical features and a significantly higher complication rate in our cohort. Although the association with prolonged hospitalization did not reach statistical significance, the higher complication burden supports the clinical relevance of this phenotype. Further validation in larger prospective studies is needed to confirm its association with clinical outcomes and to support the ongoing evolution toward phenotype-oriented management approaches.

## Data Availability

The datasets generated and/or analysed during the current study are available in the figshare repository, https://doi.org/10.6084/m9.figshare.30565802.
