# Supplementary material for: Identification of clinical phenotypes and prediction model for the mixed-infection phenotype of pediatric community-acquired pneumonia based on unsupervised machine learning
Source: Front Pediatr. 2026 May 21;14:1785262. doi: 10.3389/fped.2026.1785262 (PMC13233698; doi:10.3389/fped.2026.1785262)
Supplement: Supplementary file 1 [file Table1.docx]

Supplementary Table S1 Missing data for clustering and model variables

| Variable Category | Variable Name | Total (N) | Missing (n) | Missing (%) |
| --- | --- | --- | --- | --- |
| Demographics | Age | 305 | 0 | 0% |
|  | Sex | 305 | 0 | 0% |
| Laboratory Markers | WBC | 305 | 0 | 0% |
|  | CRP | 305 | 0 | 0% |
|  | PCT | 305 | 0 | 0% |
|  | LDH | 298 | 7 | 2.30% |
|  | D-dimer | 289 | 16 | 5.20% |
|  | Neutrophil-to-lymphocyte ratio | 305 | 0 | 0% |
|  | Lymphocyte-to-neutrophil ratio | 305 | 0 | 0% |
| Microbiological Evidence | MP infection | 305 | 0 | 0% |
|  | Viral infection | 305 | 0 | 0% |
|  | Bacterial infection | 305 | 0 | 0% |
|  | Mixed infection | 305 | 0 | 0% |
| Imaging Findings | CT consolidation | 298 | 7 | 2.30% |
|  | CT ground-glass opacity | 298 | 7 | 2.30% |
|  | Number of affected lobes | 298 | 7 | 2.30% |
