# Supplementary material for: Identification of clinical phenotypes and prediction model for the mixed-infection phenotype of pediatric community-acquired pneumonia based on unsupervised machine learning
Source: Front Pediatr. 2026 May 21;14:1785262. doi: 10.3389/fped.2026.1785262 (PMC13233698; doi:10.3389/fped.2026.1785262)
Supplement: Supplementary file 2 [file Table2.docx]

Supplementary Table S2 Characteristics of clusters obtained after removing inflammatory markers

| **Cluster** | **N** | **MP+**  **(%)** | **Viral+**  **(%)** | **Bacterial+**  **(%)** | **Mixed+**  **(%)** | **Age**  **(median, years)** | **CT consolidation**  **(%)** | **CT GGO**  **(%)** |
| --- | --- | --- | --- | --- | --- | --- | --- | --- |
| 1 | 188 | 78.7 | 4.8 | 0.0 | 16.5 | 7.0 | 94.1 | 1.1 |
| 2 | 59 | 30.5 | 11.9 | 5.1 | 44.1 | 8.0 | 44.1 | 11.9 |
| 3 | 58 | 0.0 | 10.3 | 63.8 | 22.4 | 4.5 | 89.7 | 8.6 |
